# Postexposure Treatment of Marburg Virus Infection

**DOI:** 10.3201/eid1607.100159

**Published:** 2010-07

**Authors:** Thomas W. Geisbert, Lisa E. Hensley, Joan B. Geisbert, Anders Leung, Joshua C. Johnson, Allen Grolla, Heinz Feldmann

**Affiliations:** Author affiliations: National Emerging Infectious Diseases Laboratories Institute, Boston, Massachusetts, USA (T.W. Geisbert, J.B. Geisbert);; US Army Medical Research Institute of Infectious Diseases, Fort Detrick, Maryland, USA (L.E. Hensley, J.C. Johnson);; Public Health Agency of Canada, Winnipeg, Manitoba, Canada (A. Leung, A. Grolla);; National Institutes of Health, Hamilton, Montana, USA (H. Feldmann)

**Keywords:** Marburg virus, filovirus, vesicular stomatitis virus, vaccine, treatment, viruses, dispatch

## Abstract

Rhesus monkeys are protected from disease when a recombinant vesicular stomatitis virus–based vaccine is administered 20–30 min after infection with Marburg virus. We protected 5/6 monkeys when this vaccine was given 24 h after challenge; 2/6 animals were protected when the vaccine was administered 48 h postinfection.

The filoviruses, Marburg virus (MBGV) and Ebola virus (EBOV), have been associated with sporadic episodes of hemorrhagic fever (HF) in Central Africa that produce severe disease and high mortality rates among infected patients (*1*). MBGV and EBOV are also considered potential biological weapons. No approved active or passive therapeutic modalities exist for filovirus infections. Although much progress has been made in developing preventive vaccines that can protect nonhuman primates against lethal challenge with MBGV and EBOV, advances in development of postexposure interventions against the filoviruses have not kept pace. Some degree of success has been achieved by using strategies that mitigate the coagulation abnormalities characterizing filoviral infection (*2*,*3*). Also, new postexposure treatment approaches, based on small interfering RNA (*4*) and antisense oligomers (*5*,*6*), have shown promising results in rodent models, but no reports have been published of evaluations of either strategy in the more stringent macaque models.

Recently, we showed the first complete postexposure protection of nonhuman primates against a filovirus by administering a live-attenuated recombinant vesicular stomatitis virus (rVSV) vaccine vector expressing the MBGV glycoprotein (GP) (VSVΔG MBGV GP) shortly after a high-dose MBGV challenge (*7**,**8*). We demonstrated that an rVSV vector, expressing the Zaire EBOV (ZEBOV) GP, protected 50% of rhesus macaques when administered shortly after a high-dose ZEBOV challenge (*9*). We further showed that an rVSV vector expressing the Sudan EBOV GP completely protected rhesus monkeys from a lethal challenge with this virus when administered shortly after exposure (*10*). All animals in these 3 studies were treated once with rVSV vectors 20–30 min after filovirus challenge. The primary question raised from these investigations is how far out treatment can be delayed before there is no survival or beneficial effect. Using a homologous VSVΔG MBGV GP vector, we have delineated a window of opportunity for treatment of MBGV-infected rhesus macaques.

## The Study

Animal research was conducted in compliance with the Animal Welfare Act and other federal statutes and regulations relating to animals and experiments involving animals and adhered to the principles stated in the Guide for the Care and Use of Laboratory Animals, National Research Council, 1996. Fifteen healthy, filovirus-seronegative rhesus macaques (each weighing 4 kg–7 kg) were randomized into 2 experimental groups of 6 monkeys per group and 3 control groups of 1 animal per group. All 15 animals were challenged by intramuscular (IM) injection with 1,000 PFU of MBGV (Musoke strain). Approximately 24 h after MBGV challenge, animals in experimental group 1 received a single IM injection of VSVΔG MBGV GP (≈2 × 10^7^ PFU) (*8*), and the animal in control group 1 received an equal dose of a VSV vector encoding a nonrelated GP (VSVΔG/LassaGPC). Approximately 48 h after MBGV challenge, animals in experimental group 2 received a single IM injection of VSVΔG MBGV GP (≈2 × 10^7^ PFU), and the animal in control group 2 received an equal dose of VSVΔG/LassaGPC. The animal in control group 3 was not treated. Blood samples for viral infectivity titration, reverse transcription–PCR (RT-PCR), hematologic analysis, serum biochemical analysis, and immunoglobulin (Ig) G were collected before MBGV challenge and on days 3, 6, 10, 14, and 31–35 after MBGV challenge.

Five of the 6 animals treated with VSVΔG/MBGV GP 24 h after MBGV challenge (animals 1, 2, 4–6) and 2 of the 6 animals treated with VSVΔG/MBGV GP 48 h after MBGV challenge (animals 7 and 10) survived (Figure; Table A1). In contrast, symptoms consistent with MBGV HF developed in 1 of the 6 macaques treated with VSVΔG/MBGV GP at 24 h (animal 3) and in 4 of the 6 animals treated with VSVΔG/MBGV GP at 48 h (animals 8, 9, 11, and 12); these included anorexia and a macular rash (Table 1). The 5 animals in which macular rash developed (animals 3, 8, 9, 11, and 12) also had plasma viremias >6.0 log_10_ PFU/mL by day 10; all 5 animals died during days 10–12 (Figure; Table 1; Table A1). Symptoms developed in control animals 1–3 consistent with MBGV HF; each had plasma viremia levels >7.0 log_10_ PFU/mL by day 10 and died on days 12, 12, and 11, respectively (Table A1).

**Figure:**
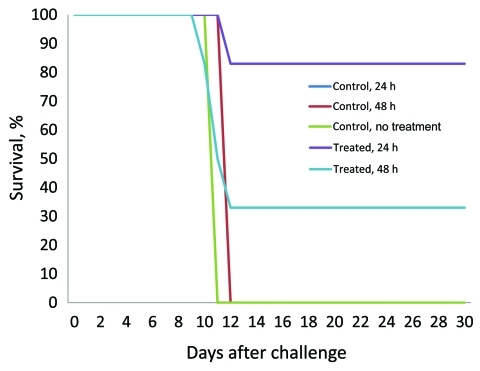
Survival curves for Marburg virus–infected rhesus macaques treated 24 or 48 h after challenge with a recombinant vesicular stomatitis virus vaccine.

**Table 1:** Viral load in rhesus monkeys after Marburg virus challenge*

Animal no.	Group	Treatment	Time of treatment after challenge, h	Plasma†				PBMC		
				Day 6	Day 10	Day 14		Day 6	Day 10	Day 14
1	Exp 1	VSV-Marburg	24	0 (–)	0 (–)	0 (–)		NT (–)	NT (+)	NT
2	Exp 1	VSV-Marburg	24	0 (–)	0 (–)	0 (–)		NT (–)	NT (+)	NT (–)
3	Exp 1	VSV-Marburg	24	3.76 (–)	6.19 (+)			NT (–)	NT (+)	
4	Exp 1	VSV-Marburg	24	0 (–)	0 (–)	0 (–)		NT (–)	NT (–)	NT (–)
5	Exp 1	VSV-Marburg	24	0 (–)	0 (–)	0 (–)		NT (–)	NT (–)	NT (–)
6	Exp 1	VSV-Marburg	24	0 (–)	0 (–)	0 (–)		NT (–)	NT (–)	NT (–)
Control 1	Cont 1	VSV-Lassa	24	3.76 (–)	7.33 (+)			NT (–)	NT (+)	
7	Exp 2	VSV-Marburg	48	0 (–)	4.20 (+)	0 (–)		NT (+)	NT (+)	NT (–)
8	Exp 2	VSV-Marburg	48	0 (–)	7.27 (+)			NT (+)	NT (+)	
9	Exp 2	VSV-Marburg	48	3.76 (–)	7.25 (+)			NT (+)	NT (+)	
10	Exp 2	VSV-Marburg	48	0 (–)	0 (–)	0 (–)		NT (–)	NT (–)	NT (–)
11	Exp 2	VSV-Marburg	48	5.24 (+)	7.35 (+)			NT (+)	NT (+)	
12	Exp 2	VSV-Marburg	48	3.76 (–)	6.81 (+)			NT (–)	NT (+)	
Control 2	Cont 2	VSV-Lassa	48	4.05 (–)	7.24 (+)			NT (+)	NT (+)	
Control 3	Cont 3	None	NA	5.07 (+)	7.15 (+)			NT (+)	NT (+)	

*PBMC, peripheral blood mononuclear cells; Exp, experimental; VSV, vesicular stomatitis virus; NT, not tested; Cont, control; (+), sample positive for Marburg virus by reverse transcription–PCR (RT-PCR); (−), sample negative for Marburg virus by RT-PCR; NA, not applicable. †Log_10_ PFU of Marburg virus per milliliter of plasma.

Two of the 6 animals that survived MBGV challenge (animals 1 and 6) showed no change in appearance or behavior that indicated overt illness. Changes in hematologic results and/or blood parameters were observed in 5 of the surviving animals (2, 4, 5, 7, and 10) during the course of the study (Table A1). Plaque assay and RT-PCR were unable to detect any evidence of MBGV in the plasma of 6 of the 7 surviving animals (1, 2, 4–6, and 10). However, RT-PCR showed evidence of MBGV in peripheral blood mononuclear cells of 2 of these surviving animals (1 and 2) at day 10 (Table 2). Viremia of 4.2 log_10_ PFU/mL developed on day 10 in 1 surviving animal (7) treated 48 h after infection, and RT-PCR showed evidence of MBGV in peripheral blood mononuclear cells of this animal on days 6 and 10. Viremia in plasma was cleared, and the animal showed little evidence of illness by day 14. The serologic response profile of MBGV infection after treatment was evaluated by IgG ELISA. All 7 animals that were treated with VSV∆G/MBGV GP and survived infection showed moderate to high levels of IgG by day 14 (320–1,000); humoral response against MBGV was not detectable in the treated animals that died or in the control animals (Table 2).

**Table 2:** Serologic response profiles of Marburg virus–infected rhesus monkeys after treatment with VSVΔG/Marburg virus glycoprotein vectors*

Animal no.	Group	Treatment	Time of treatment after challenge, h	Serum anti–Marburg virus IgG†		
				Day 6	Day 10	Day 14
1	Exp 1	VSV-Marburg	24	0	320	1,000
2	Exp 1	VSV-Marburg	24	0	100	1,000
3	Exp 1	VSV-Marburg	24	0	0	NA
4	Exp 1	VSV-Marburg	24	0	100	320
5	Exp 1	VSV-Marburg	24	0	1,000	1,000
6	Exp 1	VSV-Marburg	24	0	320	320
Control 1	Cont 1	VSV-Lassa	24	0	0	NA
7	Exp 2	VSV-Marburg	48	0	320	1,000
8	Exp 2	VSV-Marburg	48	0	0	NA
9	Exp 2	VSV-Marburg	48	0	0	NA
10	Exp 2	VSV-Marburg	48	0	320	1,000
11	Exp 2	VSV-Marburg	48	0	0	NA
12	Exp 2	VSV-Marburg	48	0	0	NA
Control 2	Cont 2	VSV-Lassa	48	0	0	NA
Control 3	Cont 3	None	NT	0	0	NA

*VSV, vesicular stomatitis virus; Ig, immunoglobulin; Exp, experimental group; Cont, control group; NA, not applicable because animal had died; NT, not treated. †Endpoint dilution titers.

## Conclusions

This rhesus macaque model represents a worse-case scenario such as an accidental needle-stick exposure of a laboratory worker or first responder to a high infectious dose of a filovirus. Accidents such as these have occurred several times over the past 5 years (*11*–*13*). Of direct relevance to our study was a recent laboratory accident in which an rVSV vector expressing the ZEBOV GP, which had been used successfully in postexposure treatment of experimentally infected nonhuman primates (*9*), was administered to a human ≈40 h after a ZEBOV needle-stick exposure (*13*). The patient received a dose of ≈5 × 10^7^ PFU of the VSV ZEBOV GP vaccine, which is consistent with doses used in nonhuman primate studies (*7*,*9*,*10*). Fever, headache, and myalgia developed in the patient hours after injection but were successfully controlled with analgesics and antipyretics. Other adverse effects were not reported, but whether treatment was effective or whether the patient never became infected remains uncertain.

MBGV infection of humans normally progresses at a slower rate than does MBGV infection of macaques, with case-fatality rates in humans of 23%–90% (*1*) suggesting that the therapeutic window may be larger for humans than for infected macaques. In addition, the challenge dose that we employed in the rhesus monkey model of MBGV HF of 1,000 PFU represents >10,000 LD_50_ doses (*14*), again showing that this is a robust challenge model. In the current study, we achieved near complete protection from death when treatment with a single-dose regimen was delayed 24 h and 33% protection when treatment was delayed 48 h postexposure. Because no approved treatments exist for exposure to infectious filoviruses, the rVSV vectors described in the current study merit consideration for treating potential exposures and for further development for human use.

## Appendix

**Table A1:** Clinical findings in rhesus monkeys infected with Marburg virus and given postexposure treatment with a recombinant VSV vector expressing the Marburg virus GP 24 h after challenge (animals 1–6) or 48 h after challenge (animals 7–12)*†

Animal no.‡	Day 6	Day 10	Day 14	Outcome
1				Survived
2		ALT↑↑↑, AST↑↑		Survived
3		Mild rash, anorexia, thrombocytopenia, lymphopenia, ALP↑, ALT↑↑↑, AST↑↑↑, GGT↑↑, TBIL↑↑↑, BUN↑↑, UA↑		Died, day 12
4		Thrombocytopenia, ALT↑↑↑, AST↑↑↑	AST↑↑	Survived
5		AST↑		Survived
6				Survived
Control 1		Fever, severe rash, anorexia, depression, lymphopenia, ALP↑, ALT↑↑↑, AST↑↑↑, GGT↑, TBIL↑, UA↑		Died, day 12
7		Thrombocytopenia, anorexia, ALT↑↑↑, AST↑↑	ALT↑↑↑	Survived
8	Fever	Mild rash, anorexia, thrombocytopenia, ALP↑, ALT↑↑↑, AST↑↑↑, GGT↑, TBIL↑↑↑, UA↑		Died, day 12
9		Moderate rash, anorexia, ALP↑, ALT↑↑↑, AST↑↑↑, GGT↑, TBIL↑↑↑		Died, day 11
10		ALT↑↑↑, AST↑↑		Survived
11		Moderate rash, anorexia, depression, ALP↑, ALT↑↑↑, AST↑↑↑, GGT↑, TBIL↑↑↑, CRE↑, UA↑		Died, day 11
12		Severe rash, anorexia, depression, thrombocytopenia, ALT↑↑↑, AST↑↑↑, GGT↑, TBIL↑↑↑, CRE↑↑, UA↑↑		Died, day 10
Control 2	Lymphopenia	Moderate rash, anorexia, depression, ALP↑, ALT↑↑↑, AST↑↑↑, GGT↑, TBIL↑↑↑		Died, day 12
Control 3		Severe rash, anorexia, depression, thrombocytopenia, ALT↑↑↑, AST↑↑↑, GGT↑, TBIL↑↑, UA↑		Died, day 11

*GP, glycoprotein; ALP, alkaline phosphatase; ALT, alanine aminotransferase, AST, aspartate aminotransferase;, GGT, gamma-glutamyl transferase; TBIL, total bilirubin;, BUN, blood urea nitrogen; CRE, creatinine; UA, uric acid; ↑, 2–3-fold increase; ↑↑, 4–5-fold increase; ↑↑↑, >5-fold increase. †Fever was defined as a temperature >2.5ºF over baseline or at least 1.5ºF over baseline and >103.5ºF. Mild rash consisted of focal areas of petechiae covering <10% of the skin; moderate rash, areas of petechiae covering 10%–40% of the skin; severe rash, areas of petechiae and/or echymosis covering >40% of the skin. Lymphopenia and thrombocytopenia were defined by a >35% drop in numbers of lymphocytes or platelets, respectively. ‡Control animals received a recombinant vesicular stomatitis virus vector expressing a nonspecific glycoprotein at 24 h (control 1) or 48 h (control 2) or were given no treatment (control 3).
